# Supplementary material for: Bayesian refinement of protein structures and ensembles against SAXS data using molecular dynamics
Source: PLoS Comput Biol. 2017 Oct 18;13(10):e1005800. doi: 10.1371/journal.pcbi.1005800 (PMC5662244; doi:10.1371/journal.pcbi.1005800)
Supplement: S1 Archive — (BZ2) [file pcbi.1005800.s011.tar.bz2 › gromacs_4.62_bayesian_saxs_refi/share/html/online/trjconv.html]

trjconv


| trjconv Main Table of Contents |

**VERSION 4.6  
Sat 19 Jan 2013**

---

### Description

trjconv can convert trajectory files in many ways:  
**1.** from one format to another  
**2.** select a subset of atoms  
**3.** change the periodicity representation  
**4.** keep multimeric molecules together  
**5.** center atoms in the box  
**6.** fit atoms to reference structure  
**7.** reduce the number of frames  
**8.** change the timestamps of the frames
(-t0 and -timestep)  
**9.** cut the trajectory in small subtrajectories according
to information in an index file. This allows subsequent analysis of
the subtrajectories that could, for example, be the result of a
cluster analysis. Use option -sub.
This assumes that the entries in the index file are frame numbers and
dumps each group in the index file to a separate trajectory file.  
**10.** select frames within a certain range of a quantity given
in an .xvg file.

The program trjcat is better suited for concatenating multiple trajectory files.

Currently seven formats are supported for input and output:
.xtc, .trr, .trj, .gro, .g96,
.pdb and .g87.
The file formats are detected from the file extension.
The precision of .xtc and .gro output is taken from the
input file for .xtc, .gro and .pdb,
and from the -ndec option for other input formats. The precision
is always taken from -ndec, when this option is set.
All other formats have fixed precision. .trr and .trj
output can be single or double precision, depending on the precision
of the trjconv binary.
Note that velocities are only supported in
.trr, .trj, .gro and .g96 files.

Option -app can be used to
append output to an existing trajectory file.
No checks are performed to ensure integrity
of the resulting combined trajectory file.

Option -sep can be used to write every frame to a separate
.gro, .g96 or .pdb file. By default, all frames all written to one file.
.pdb files with all frames concatenated can be viewed with
rasmol -nmrpdb.

It is possible to select part of your trajectory and write it out
to a new trajectory file in order to save disk space, e.g. for leaving
out the water from a trajectory of a protein in water.
**ALWAYS** put the original trajectory on tape!
We recommend to use the portable .xtc format for your analysis
to save disk space and to have portable files.

There are two options for fitting the trajectory to a reference
either for essential dynamics analysis, etc.
The first option is just plain fitting to a reference structure
in the structure file. The second option is a progressive fit
in which the first timeframe is fitted to the reference structure
in the structure file to obtain and each subsequent timeframe is
fitted to the previously fitted structure. This way a continuous
trajectory is generated, which might not be the case when using the
regular fit method, e.g. when your protein undergoes large
conformational transitions.

Option -pbc sets the type of periodic boundary condition
treatment:  
\* mol puts the center of mass of molecules in the box,
and requires a run input file to be supplied with -s.  
\* res puts the center of mass of residues in the box.  
\* atom puts all the atoms in the box.  
\* nojump checks if atoms jump across the box and then puts
them back. This has the effect that all molecules
will remain whole (provided they were whole in the initial
conformation). **Note** that this ensures a continuous trajectory but
molecules may diffuse out of the box. The starting configuration
for this procedure is taken from the structure file, if one is
supplied, otherwise it is the first frame.  
\* cluster clusters all the atoms in the selected index
such that they are all closest to the center of mass of the cluster,
which is iteratively updated. **Note** that this will only give meaningful
results if you in fact have a cluster. Luckily that can be checked
afterwards using a trajectory viewer. Note also that if your molecules
are broken this will not work either.  
The separate option -clustercenter can be used to specify an
approximate center for the cluster. This is useful e.g. if you have
two big vesicles, and you want to maintain their relative positions.  
\* whole only makes broken molecules whole.

Option -ur sets the unit cell representation for options
mol, res and atom of -pbc.
All three options give different results for triclinic boxes and
identical results for rectangular boxes.
rect is the ordinary brick shape.
tric is the triclinic unit cell.
compact puts all atoms at the closest distance from the center
of the box. This can be useful for visualizing e.g. truncated octahedra
or rhombic dodecahedra. The center for options tric and compact
is tric (see below), unless the option -boxcenter
is set differently.

Option -center centers the system in the box. The user can
select the group which is used to determine the geometrical center.
Option -boxcenter sets the location of the center of the box
for options -pbc and -center. The center options are:
tric: half of the sum of the box vectors,
rect: half of the box diagonal,
zero: zero.
Use option -pbc mol in addition to -center when you
want all molecules in the box after the centering.

It is not always possible to use combinations of -pbc,
-fit, -ur and -center to do exactly what
you want in one call to trjconv. Consider using multiple
calls, and check out the GROMACS website for suggestions.

With -dt, it is possible to reduce the number of
frames in the output. This option relies on the accuracy of the times
in your input trajectory, so if these are inaccurate use the
-timestep option to modify the time (this can be done
simultaneously). For making smooth movies, the program g\_filter
can reduce the number of frames while using low-pass frequency
filtering, this reduces aliasing of high frequency motions.

Using -trunc trjconv can truncate .trj in place, i.e.
without copying the file. This is useful when a run has crashed
during disk I/O (i.e. full disk), or when two contiguous
trajectories must be concatenated without having double frames.

Option -dump can be used to extract a frame at or near
one specific time from your trajectory.

Option -drop reads an .xvg file with times and values.
When options -dropunder and/or -dropover are set,
frames with a value below and above the value of the respective options
will not be written.

### Files

| option | filename | type | description |
| --- | --- | --- | --- |
| **-f** | traj.xtc | Input | Trajectory: xtc trr trj gro g96 pdb cpt |
| **-o** | trajout.xtc | Output | Trajectory: xtc trr trj gro g96 pdb |
| **-s** | topol.tpr | Input, Opt. | Structure+mass(db): tpr tpb tpa gro g96 pdb |
| **-n** | index.ndx | Input, Opt. | Index file |
| **-fr** | frames.ndx | Input, Opt. | Index file |
| **-sub** | cluster.ndx | Input, Opt. | Index file |
| **-drop** | drop.xvg | Input, Opt. | xvgr/xmgr file |

### Other options

| option | type | default | description |
| --- | --- | --- | --- |
| **-[no]h** | bool | no | Print help info and quit || **-[no]version** | bool | no | Print version info and quit || **-nice** | int | 19 | Set the nicelevel || **-b** | time | 0 | First frame (ps) to read from trajectory || **-e** | time | 0 | Last frame (ps) to read from trajectory || **-tu** | enum | ps | Time unit: fs, ps, ns, us, ms or s || **-[no]w** | bool | no | View output .xvg, .xpm, .eps and .pdb files || **-xvg** | enum | xmgrace | xvg plot formatting: xmgrace, xmgr or none || **-skip** | int | 1 | Only write every nr-th frame || **-dt** | time | 0 | Only write frame when t MOD dt = first time (ps) || **-[no]round** | bool | no | Round measurements to nearest picosecond || **-dump** | time | -1 | Dump frame nearest specified time (ps) || **-t0** | time | 0 | Starting time (ps) (default: don't change) || **-timestep** | time | 0 | Change time step between input frames (ps) || **-pbc** | enum | none | PBC treatment (see help text for full description): none, mol, res, atom, nojump, cluster or whole || **-ur** | enum | rect | Unit-cell representation: rect, tric or compact || **-[no]center** | bool | no | Center atoms in box || **-boxcenter** | enum | tric | Center for -pbc and -center: tric, rect or zero || **-box** | vector | 0 0 0 | Size for new cubic box (default: read from input) || **-clustercenter** | vector | 0 0 0 | Optional starting point for pbc cluster option || **-trans** | vector | 0 0 0 | All coordinates will be translated by trans. This can advantageously be combined with -pbc mol -ur compact. || **-shift** | vector | 0 0 0 | All coordinates will be shifted by framenr\*shift || **-fit** | enum | none | Fit molecule to ref structure in the structure file: none, rot+trans, rotxy+transxy, translation, transxy or progressive || **-ndec** | int | 3 | Precision for .xtc and .gro writing in number of decimal places || **-[no]vel** | bool | yes | Read and write velocities if possible || **-[no]force** | bool | no | Read and write forces if possible || **-trunc** | time | -1 | Truncate input trajectory file after this time (ps) || **-exec** | string |  | Execute command for every output frame with the frame number as argument || **-[no]app** | bool | no | Append output || **-split** | time | 0 | Start writing new file when t MOD split = first time (ps) || **-[no]sep** | bool | no | Write each frame to a separate .gro, .g96 or .pdb file || **-nzero** | int | 0 | If the -sep flag is set, use these many digits for the file numbers and prepend zeros as needed || **-dropunder** | real | 0 | Drop all frames below this value || **-dropover** | real | 0 | Drop all frames above this value || **-[no]conect** | bool | no | Add conect records when writing .pdb files. Useful for visualization of non-standard molecules, e.g. coarse grained ones |

---

http://www.gromacs.org  
gromacs@gromacs.org
